# Chronaxie Measurements in Patterned Neuronal Cultures from Rat Hippocampus

**DOI:** 10.1371/journal.pone.0132577

**Published:** 2015-07-17

**Authors:** Shani Stern, Andres Agudelo-Toro, Assaf Rotem, Elisha Moses, Andreas Neef

**Affiliations:** 1 Department of Physics of Complex Systems, Weizmann Institute of Science, Rehovot, Israel; 2 Department of Non-linear Dynamics, Max Planck Institute for Dynamics and Self-Organization and Bernstein Group ‘Biophysics of Neural Computation’, Göttingen, Germany; 3 Department of Physics and School of Engineering and Applied Sciences, Harvard University, Cambridge, Massachusetts, United States of America; Osaka University Graduate School of Medicine, JAPAN

## Abstract

Excitation of neurons by an externally induced electric field is a long standing question that has recently attracted attention due to its relevance in novel clinical intervention systems for the brain. Here we use patterned quasi one-dimensional neuronal cultures from rat hippocampus, exploiting the alignment of axons along the linear patterned culture to separate the contribution of dendrites to the excitation of the neuron from that of axons. Network disconnection by channel blockers, along with rotation of the electric field direction, allows the derivation of strength-duration (SD) curves that characterize the statistical ensemble of a population of cells. SD curves with the electric field aligned either parallel or perpendicular to the axons yield the chronaxie and rheobase of axons and dendrites respectively, and these differ considerably. Dendritic chronaxie is measured to be about 1 ms, while that of axons is on the order of 0.1 ms. Axons are thus more excitable at short time scales, but at longer time scales dendrites are more easily excited. We complement these studies with experiments on fully connected cultures. An explanation for the chronaxie of dendrites is found in the numerical simulations of passive, realistically structured dendritic trees under external stimulation. The much shorter chronaxie of axons is not captured in the passive model and may be related to active processes. The lower rheobase of dendrites at longer durations can improve brain stimulation protocols, since in the brain dendrites are less specifically oriented than axonal bundles, and the requirement for precise directional stimulation may be circumvented by using longer duration fields.

## Introduction

Novel intervention technologies for the brain such as Deep Brain Stimulation (DBS) and Transcranial Magnetic Stimulation (TMS) can activate or inhibit neurons deep inside brain regions, thus possibly affecting a specific function or pathology [1–4]. The potential for further advances in such neuromodulatory techniques depends critically on understanding the interaction of neurons with an externally imposed electric field [5–8].

It is usual to attribute the initiation of the action potential (AP) during super-threshold stimulation of central nervous system (CNS) neurons to the axon: the segments with the highest sodium channel density, the axon initial segment and the nodes of Ranvier are likely the specific targets of stimulation [9–11]. It is furthermore natural to assume that axons, which are typically longer than dendrites, are easier to excite in a uniform externally imposed electric field since their relative length allows larger depolarization. In regions where the axons are oriented, e.g. in nerve fibers, the efficacy of the stimulation would depend on the orientation with respect to the stimulus field. However, over the past years several authors have shown the possibility of initiation of APs in the dendrites, and have emphasized their importance for the generation of action potentials. While only representing 0.4 km of cable compared to 4 km of axons in a typical 1 mm^3^ cube of cortex [12], dendrites are important information processors and a relevant target for stimulation. Golding [13] showed that locally generated dendritic spikes trigger potentiation of distal dendrites, and therefore contribute to learning mechanisms. The role of dendrites in initiating APs is also highlighted by the discovery of sodium and calcium mediated spikes originating in them [14–18]. Compared to axons, dendrites tend to extend in all directions over the cortex making them the ideal target for stimulation under an externally imposed uniform electric field.

As pointed out already by Ranck [19], a powerful tool for evaluating the contribution of each part of the cell to initiation of the AP is the strength-duration curve [20–22], which plots the minimal amplitude of the electric field pulse needed for excitation versus the pulse’s duration. Since the amplitude will decrease like an exponential with the duration [21] a time constant called *chronaxie* can usually be extracted, giving the typical duration of the stimulus needed to induce an AP. The strength-duration curve is fully described by only two parameters—the chronaxie along with the *rheobase*, which is the asymptotic value of the strength (or amplitude) needed for excitation with a very long or ‘infinite’ pulse. For a passive, electrotonically compact cell the chronaxie, as defined by Lapicque and Weiss, is proportional to the membrane time constant [23] with the factor *ln*2. However this simple relation breaks down if the neurons are spatially extended and a more realistic description such as the cable equation must be employed. Other time constants then play a more dominant role [24, 25] and chronaxies can be much shorter than the membrane time constant.

It is therefore not obvious how the chronaxie, as measured from the strength-duration curve, is related to the passive membrane properties and the morphology of the cell, and in fact several discrepancies and controversies have arisen. Contemporary estimates of 1–10 ms time constants of soma and dendrites were considered far above the 0.05–0.2 ms chronaxies observed during micro-stimulation of CNS, i.e. local stimulation with micro-electrodes. The difference was taken as an indication that only axons are involved in the initiation of APs [19]. Still, an explanation for higher, commonly observed 0.2–1 ms chronaxies in gray matter was not found. Intracellular chronaxies were estimated to be around 15 ms [10, 26]. Furthermore, while the role of dendrites in AP initiation during micro stimulation has been investigated in numerical simulations [11], little is known about the role of dendrites during macro-stimulation. Experimentally it is also a technologically challenging problem to separate the different contributions of axons and dendrites to the initiation of stimulation.

Numerical simulation and modeling have played a significant role in elucidating the bone of contention for determining the origin of AP initiation. Chronaxies for intracellular and extracellular stimulation depend on the geometrical properties of the cell [27, 28] while the membrane time constant does not. Modeling shows that chronaxies for intracellular stimulation should be at least twice as large as those during extracellular micro-stimulation, which is still far from the experimentally observed differences. These efforts provide a refreshing view on the effects of the extracellular field but also leave open several questions. For example, it is still not known whether the larger chronaxie observed in grey matter (>200 μs) can be explained by geometrical differences. Similarly, it remains unclear if other elements of the neuronal cell such as the dendrites participate in the overall response of CNS tissue to extracellular stimulation, nor is it obvious how micro-stimulation observations relate to macro-stimulation.

In this study we selectively stimulated either only dendrites or dendrites and axons together, using patterned cultures. This allows for separation into measurements of axonal population and dendritic population. We have previously shown that neuronal networks cultured from hippocampal neurons serve as a good model for understanding the activation of neurons during macro-stimulation [29] and in particular, orientation of the culture along one-dimensional strips allows a corresponding orientation of the axons [30]. Since the electric field is also oriented, this allows in the present work for a separation of the response of axons from the dendrites employing the property that cables parallel to the field are more easily stimulated than cables perpendicular to it [31, 32]. Surprisingly, we show here that while axons are more excitable at short duration pulses, dendrites respond better at long pulses, with the crossover occurring at about one millisecond.

We then propose a passive cable model that starts with simply structured neurons and goes on to reconstructed neurons, where we elucidate the influence of morphology and membrane properties on the chronaxie. This explains the observed dendritic chronaxies. It cannot, however explain the short chronaxie and higher rheobase of the axons, and we conclude with the suggestion that this is due to active processes.

## Materials and Methods

All procedures were approved by the Weizmann Institutional Animal Care and Use Committee.

### Primary cultures

Primary cultures were prepared from rat hippocampi of 19-day-old embryos taken from Wistar rats. Two-dimensional (2D) cultures were plated at 850,000 cells per ml, while one-dimensional cultures were plated at 650,000 cells per ml. The full description is given in S1 Text. Images of a developing 1D culture can be found in S1 Fig.

### Calcium imaging

Calcium imaging (CI) was used to measure the response of the entire culture to stimulation, using fluo-4AM calcium indicator. The full protocol is described in S2 Text.

### Electric stimulation

**Fig 1:**
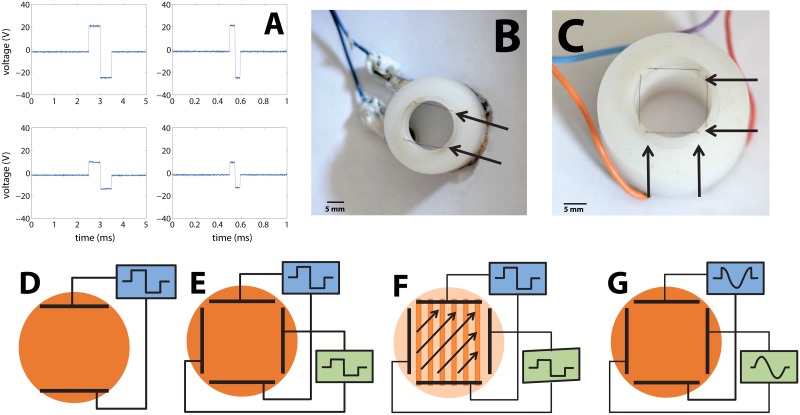
Apparatus used to stimulate neuronal cultures with external electric fields. (A) Waveforms of the measured applied voltage between the electrodes (upper row) and within the fluid (lower row), approximately at the center of the experimental cell where the culture is located. Left column is for pulse durations of 1ms and the right is for durations of 100 μs. (B) The plastic insert with one pair of electrodes used to stimulate the neuronal culture. The electrode wires are indicated in the picture by arrows. A plastic rim extending into the medium locates the two parallel platinum wire electrodes about 1 mm above the culture. (C) The device used to stimulate the neuronal culture with two orthogonal pairs of electrodes. A plastic rim holds two pairs of platinum electrodes about 1mm above the culture, which are denoted in the picture by arrows. (D) Sketch of the electrical circuit for a pair of electrodes that is driven by a single square pulse with varying durations. The electric field created between these electrodes is used to stimulate a neuronal network cultured on a glass coverslip. (E, F) Sketch of the electrical circuit for two pairs of electrodes that are fed with separate single square pulses with varying durations (synchronized but with no common ground). Changing the relative amplitudes changes the orientation of the electrical field, which is used to directionally stimulate a neuronal network cultured on a cover slip. (G) Sketch of the electrical circuit for obtaining a rotating electric field. Two pairs of electrodes are each fed with one cycle of sine or of cosine voltage pulses (i.e. two signals with the same amplitude but phase shifted by π/2).

Electric stimulation was achieved using bath electrodes made of two parallel platinum wires (0.005'' thick; A-M Systems, Carlsborg WA, Catalog number 767000) 11 mm long and 13 mm apart that were immersed in the recording dish 1mm above the 13 mm diameter cover glass with the culture. For stimulation one cycle of a bipolar square pulse was used (50% duty cycle, see Fig 1A). The duration of the single cycle was between 0.01 and 4 ms and with maximal amplitude of ±22 V in 5 of 9 cultures, and ±30 V in the rest. The electrical field induced was orthogonal to the electrode wire and produced a maximum electric field of about 0.9 V/mm (see measurements in S2 Fig). Electrodes were embedded in a plastic rim (see Fig 1B and 1C), which positioned the electrodes inside the petri dish above the cover slip on which the culture grows. Adding a second, perpendicular pair of electrodes (Fig 1C) enabled us to give more complex field signals). A full description of the setup is given in S3 Text.

### Pharmacology: 4-AP experiments (on connected two dimensional networks)

4-Aminopyridine (4-AP) is a channel blocker, considered to be the standard drug for the inhibition of transient “A currents”. The A-type potassium current prevents initiation and back-propagation of action potentials into the dendrites [33]. While at the high concentration of 8 mM 4-AP influences a wide variety of K+ channels, at 2 mM it was shown to be selective for the transient (fast) K+ channels [33, 34]. More details are given in S4 Text.

### Pharmacology: Network disconnection for the strength-duration curve (one dimensional networks)

Individual neuron responses were measured by disconnecting the network, blocking synaptic transmission by the combined application of 10 μM of the NMDA receptor antagonist 2-amino-5 phosphonovaleric acid (Sigma-Aldrich), 40 μM GABA receptors antagonist bicuculline-methochloride (Sigma-Aldrich) and 10 μM of the AMPA/kainate receptor antagonist 6-cyano-7-nitroquinoxaline-2,3-dione (CNQX, Sigma-Aldrich). Details are given in S5 Text.

### Deriving the strength-duration relation: Experimental setup

1D cultures are a good tool to estimate chronaxie and rheobase, because they allow to differentiate between axons and dendrites. We previously showed [35] that axons align with the pattern of the network and that dendrites have no preferred orientation. We used patterns of long thin lines (170 μm wide, 11 mm long) resulting in axons growing in one specific direction. Inducing an electrical field parallel to this orientation will excite both axons and those dendrites that are aligned with the patterned line. An electrical field orthogonal to this direction will not cause excitation of axons since they are perpendicular to this field. It can, however, cause stimulation of dendrites, since dendrites are isotropically oriented. The variety of possible experimental configurations and orientations that were used are presented in Fig 1. To obtain both parallel and perpendicular orientations of the electric field without physically rotating the culture, two orthogonal sets of electrodes were used (Fig 1C). By tuning the relative strength of the fields in each of the two pairs of electrodes, any angle between 0 and 360° could be obtained.

### Deriving the strength-duration relation: Population response in a disconnected network

Although connected cultures provide information about the orientation selectivity of axons and dendrites, the behavior is typically dominated by a small fraction of the most excitable cells. These cells require lower durations and amplitudes for stimulation, will fire first and trigger a network response via synaptic communication. Chemical disconnection of the cultures changes this by removing network effects and can provide more concise information about the role of cell morphology in stimulation. Network disconnection gives us statistical information about the response of all the cells in the culture to the electric field. In general, it is expected that disconnected cultures will require a significantly higher duration/amplitude for excitation than connected ones, determined by the typical or average excitability of the neuron population rather than by a small percentage of highly excitable neurons. All samples were measured both with and without synaptic blockers, and this expectation was indeed verified.

**Fig 2:**
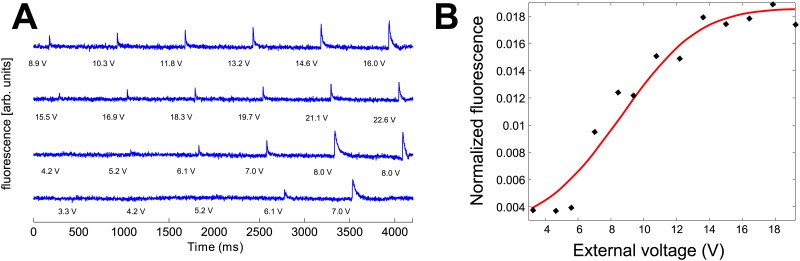
Measuring population threshold from fluorescence intensity as a function of the field strength for a given duration. (A) Fluorescence intensity measured using calcium imaging, from about 100 neurons in the 1D culture that are within the field of view of the microscope. CNQX, APV and bicuculline were used to completely disconnect the network. External stimulation was given at times marked by the vertical grey lines. The four blue curves show typical fluorescence responses of the network, with excitation seen as a sharp increase in fluorescence intensity. The number adjacent to the grey line represents the amplitude of the stimulating signal (in volts). (B) At a fixed pulse duration, the intensity of the normalized fluorescence vs. voltage used for stimulation is a cumulative Gaussian distribution. The mean minimal amplitude needed for stimulation (“Strength”) was obtained by fitting each experimentally measured fluorescence intensity as a function of the electric field amplitude to a cumulative Gaussian distribution (the Error function) and extracting the expectation value of the Gaussian, The half maximum of this curve is thus taken as the threshold for excitation of the neuronal population.

To measure chronaxie for a population of disconnected neurons we assumed that the voltage threshold in a given pulse duration for excitation of neurons in the culture is distributed normally. When applying a pulse with a given voltage and duration, all neurons with a threshold for activation below this strength/duration combination will fire. The distribution of the number of neurons that fired in response to a given voltage is therefore a cumulative Gaussian distribution, i.e. an Error function (see Fig 2). We measure the number of neurons that fired for a given voltage/duration by means of fluorescence amplitude, relying on proportionality between fluorescence intensity and the amount of spikes fired. We can then characterize the population response by the expectation and variance of the measured fluorescence amplitude distributed as an Error function. The expectation of the amplitude represents the voltage for which half of the neurons will respond, or the mean voltage for firing in the culture.

### Deriving the strength-duration relation: Parallel and perpendicular orientations

The projection of the axons along the line in linear cultures has been previously shown to be on average 85% of their length [35]. The lines in our apparatus are quasi-1D with a width of 170 μm, much less than their length of 10 mm. We previously showed that the typical axon length in such a geometry is between 500 to 1000 μm long, and that the geometric limitations of the culture make them follow the largest dimension of the line [35]. Boundary conditions in a finite-cable-like neuronal process determine that the largest effect is obtained when the field is aligned with the cable, and that this effect is reduced to essentially zero when the field is perpendicular [19, 36, 37]. Knowing that the axons tend to be oriented along the semi 1D bars allows to separate the axonal response and to compare it with dendritic response. Dendrites of neurons grown on lines typically have a shorter length of about 200 μm, so that they can grow in all directions and are isotropic [30].

To measure the chronaxie and rheobase, we applied pulses with varying amplitudes of voltage and varying pulse durations, and then looked for the minimal voltage that elicited excitation in 50% of the neurons in the disconnected network at every pulse duration. We measured the response of each culture in at least two different conditions: parallel (exciting axons and dendrites) and orthogonal to the lines (exciting dendrites only). Since dendrites and axons have different morphology and different ion channel concentrations, their chronaxie and rheobase are different. This difference enables the separation of axons and dendrites in the data.

Amplitude strength was normalized by the response threshold at long durations (2 ms) for every culture measured V_norm_ = V/V(2ms). This normalization gives similar conditions to cultures which have different neurite lengths. This is particularly important when the cultures are mature and axon length can vary. It is known that age can influence the culture in several ways [38]. In our case the age of the culture correlates with shorter excitation duration of the parallel excitation direction, but not for the perpendicular one, a fact which we ascribe to the growth of longer axons as a function of age. In the Strength Duration experiments the average age of the cultures was DIV = 44±8 (n = 7), which is long compared to the other experiments we conducted. The reason is that after network disconnection the culture responds on average to longer durations. Therefore young cultures needed relatively long durations and were less reliable and harder to measure. In a connected network the most sensitive neurons are the ones that respond, so shorter durations are in fact useful. Unless otherwise stated, here and in statistical data given below, error estimates provided are the standard error SE.

### Deriving the strength-duration relation: Extracting the chronaxie

In the presence of an electric field the nerve fiber accumulates charge on its edges like a capacitor, and following analysis by [39], upon application of an external voltage V_0_, membrane potential will build according to the following equation:

$$V\left(t\right)=V_{0}\left[1-\text{exp}\left(\frac{-t}{\tau }\right)\right]$$ (1)

This equation suggests a single time constant for accumulating charge in the membrane. To extract this time constant, we assume that the membrane potential V(t) will continue increasing until a time t* when it reaches the value of the potential needed for crossing the threshold for excitation (V_rh_), at which stage it will fire:

$$V_{0}\left[1-\text{exp}\left(\frac{-t^{*}}{\tau }\right)\right]=V_{rh}$$ (2)

Therefore, for a given pulse duration t, the minimal external voltage that can be applied to create excitation is given by:

$${\text{V}}_{0}=\frac{V_{rh}}{1-\text{exp}\left(\frac{-t}{\tau }\right)}$$ (3)

or similarly:

$${\text{V}}_{0}=\frac{V_{rh}}{1-2^{\frac{-t}{C}}}$$ (4)

where C denotes the Chronaxie, and:

C=τln(2) (5)

Fitting the strength duration curve to an equation of V_0_ vs. t has been shown to be a particularly powerful way for constraining the data. Since the SD curve is fully determined by the chronaxie and V_rh_, with just two parameters all the strength-duration measurements for a given neuronal system can be described [21,22]. Practically, the graph is well characterized by the external voltage needed for exciting the culture at a very long duration field (rheobase) and the charging time constant (chronaxie).

**Fig 3:**
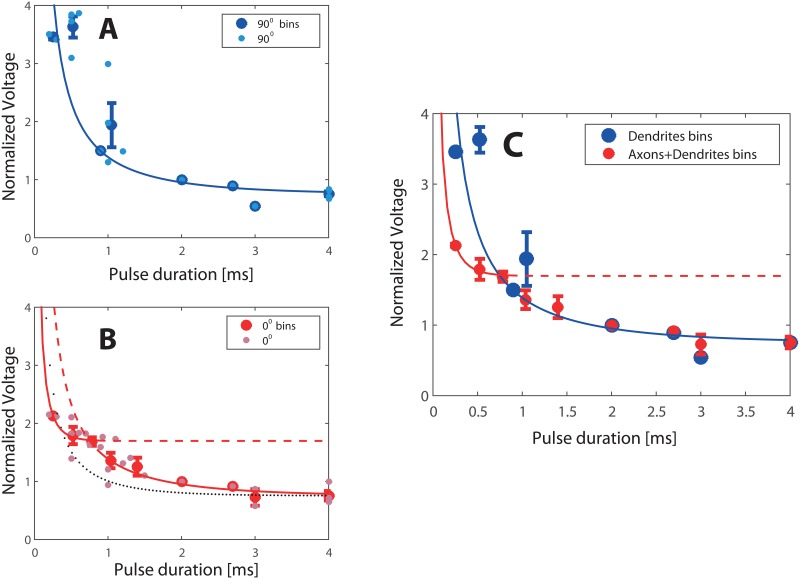
Measured Strength-Duration (SD) curves. Strength was measured as a population response (see Methods). A total of n = 33 experiments are presented. The linear cultures were grown on 9 different dishes, each dish contained 5 lines and each line was typically stimulated for 3 or 4 different pulse durations. 1D cultures were stimulated first at 0° for different durations and of these n = 25 were also stimulated at 90°. All dishes were stimulated at 2ms to allow normalization (see Methods). (A) Voltage needed to excite the culture (‘Strength’) is plotted as a function of the pulse duration, with the field at 90° to the line. Small light blue filled circles denote measured data; large blue circles denote binned data. The blue line is the best fit with τ = (1.3±0.4) ms, as described in the text. (B) Strength-Duration with the field parallel (0°) to the line of the culture. Small pink squares denote individual measured data points; red large squares denote binned data. Black dotted line is a fit with a single time constant. The two red lines are obtained using a fit with two time constants; For durations shorter than 1 ms: τ = (160±20) μs, and above 1 ms: τ = (1.4±0.2) ms. The curve for the long durations almost completely merges with the 90° curve, indicating that indeed the same mechanisms are active as in the dendrites stimulation. (C) Data from electric field orientation with respect to the linear culture at 0° and 90° together. From the 90° curve we derive the time constant of the dendrites. The 0° curve diverges from the 90° curve at short pulses. From the data in short pulses we derive the time constant of the axons. Dendrites can be seen to be more sensitive to excitation at pulses longer than 1 ms, with a Rheobase of 0.75 Vnorm compared to 1.7 Vnorm of the axons, while axons are more sensitive to excitation at pulses shorter than 1 ms.

Scatter in the data for the excitation was eliminated by averaging and binning the data. Thus the plots presented in Fig 3A and 3B show the original raw data along with the binned results, while for creating the SD curve in Fig 3C we used the binned, averaged data only. Binning was performed in 300μs intervals and the binned data points were used to produce a Strength-Duration curve and for fitting its parameters to the plots.

### Numerical simulations

To understand the response of the cultured cell, computational models of neurites (cables) and neurons under stimulation were developed. Model cells consisted of concatenated cylinders, which were idealized to 1D cables. Stimulation of a cylinder under a homogenous field corresponds to the injection of currents with opposite amplitudes at the two end of the cylinder. The source terms that were added to the cable equation on the right hand side are proportional to the projection of the spatial derivative of the electric field along the cable *E*_*l*_:

$$\lambda^{2}\frac{\partial^{2}V}{\partial l^{2}}-\tau_{m}\frac{\partial V}{\partial t}-V=\lambda^{2}\frac{\partial E_{l}}{\partial l}$$ (6)

as described earlier [29]. The parameters are the axial length constant λ, and time constant τ_m_. The cable ends are assumed to be sealed, implying the boundary conditions:

$$\frac{\partial V\left(0,t\right)}{\partial l}=\frac{\partial V\left(L,t\right)}{\partial l}=0$$ (7)

Source terms were calculated using a Python custom program according to the predefined temporal waveform and direction of the electric field. The actual simulation was then performed with the IClamp method of the simulation environment NEURON [40]. The result of such a simulation is a family of voltage waveforms V(t), one for each point in the neuronal tree. These time trajectories reflect the expected voltage changes in the experiment *for stimuli that create a subthreshold depolarization* i.e. polarizations in a range where a passive behavior without changes in ion channel activity can be expected. The simulation of *passive* membrane charging is useful for studying the strength-duration relation if the ion channel activation leading to active, all-or-none excitation occurs only once a certain threshold potential is crossed, and this activation then occurs within a narrow voltage region above the subthreshold range. In this case the passive strength duration curve gives a reliable estimate of the strength duration curves for full excitation. Extraction of the chronaxies from the cable simulations and the parameters used are specified in S6 Text.

## Results

We examine the relation of dendritic to axonal excitation with both disconnected and connected cultures. We first use disconnected 1D patterned cultures to measure strength-duration curves for stimulation of axons and dendrites versus stimulation of only dendrites. This allows us to extract the population averaged chronaxie of axons and of dendrites. We then look at connected networks as a model that is closer to neuronal networks in the brain: 1D networks relate to nerve fibers while 2D networks relate to areas where axons have a more random orientation. By rotating the direction between field and axons we show that connected 2D networks respond isotopically while the response of connected 1D networks is strongly oriented. This is ascribed to the orientation of axons, which dominate the sensitivity to stimulation at short times. Finally, we examine the role of active processes by using 4-AP to block A-type potassium channels and discriminate axonal from dendritic excitation mechanisms. Theory and numerical simulations are implemented to suggest an explanation for the observed chronaxie of dendrites.

### Measuring strength-duration relations and obtaining separate chronaxies for axons and for dendrites

To separate between axonal and dendritic stimulation, we used neuronal cultures grown in quasi one-dimensional (1D) patterns, since the axons here are typically directed along the line of the culture. One-dimensional culture dishes were chemically disconnected with the synaptic blockers CNQX, APV and bicuculline, following the procedure described in the Methods section. Fig 3 presents the strength-duration (SD) relationship (see Methods) for n = 9 cultures stimulated with different pulse durations at 0° and 90° angles with respect to the 1D pattern. The half-maximum value of the cumulative Gaussian distribution of fluorescence intensity vs. stimulating voltage at each pulse duration gives the voltage at which half of the cells responded (see Methods, Fig 2). This value statistically represents the excitation threshold of the culture.

In Fig 3 the SD data at parallel and perpendicular orientations shows a clear difference between the axonal and dendritic excitation at short pulses. While it is possible to induce firing by dendritic stimulation (at 90°) with short stimuli (around 500 μs), it is relatively difficult. In fact, it requires roughly twice the amplitude needed to produce axonal + dendritic stimulation (at 0°). Notable and surprising is the fact that at long durations both curves converge, implying a similar rheobase.

Looking at the SD curves, we notice that, after normalization by the amplitude at 2 ms, both 90° and 0° curves asymptote to about 0.75 V_norm_. Since the SD curve is totally determined by chronaxie and rheobase (see Methods), we fix the rheobase at this value of V_rh_ = 0.75 V_norm_ for both directions (0° and 90°), and apply a fit only for the time constant. As shown in Fig 3A, this works well for the 90° data, giving a time constant τ = (1.3±0.45) ms with an R^2^ value of about 0.7. Here and in the fitting below errors given are the 95% confidence intervals. This is equivalent to a value of the chronaxie for dendrites of 0.9 ms. However, the approach fails when we turn to the 0° data, as shown in Fig 3B. The R^2^ value for the fit (black curve) drops down to 0.5, and misses most of the data points.

The solution is found by noticing that if we separate the time duration t of the pulse into two regimes, t>1ms and t<1ms, a very good fit can be obtained for each of these regimes separately. Moreover, the fit at t>1ms is very similar to that of the 90° fit both for the rheobase and for the time constant. As shown in Fig 3B (red curves), the data is well fit by τ = (1.4±0.2) ms for t>1ms and by τ = (160±20)μs for t<1ms, with R^2^ values greater than 0.9. These correspond to chronaxies of 0.9 ms for t>1 ms and of 110 μs for t<1 ms. Note that while the rheobase is still constrained at V_rh_ = 0.75 V_norm_ for t>1ms, at t<1 ms the separate fit necessitated a rheobase that was best fit as V_rh_ = 1.7 V_norm_. The agreement of the fits for t>1 ms between the 0° and 90° curves seen in (Fig 3C) shows that for stimulus durations above 1 ms of the SD curves for dendrites alone (90°) and those for axons and dendrites combined (0°) coincide. This strongly suggests a common source of excitation at long pulse durations, namely the dendritic compartment, and indicates that the same dendritic excitation mechanism dominates in this regime. In contrast, at pulse durations t<1 ms it is the axons that are excited first and cause the neuron to fire. We conclude that the response to long duration pulses is dominated by the dendritic contribution, while for the shorter durations the stimulation is mainly axonal. Because dendrites have a lower rheobase than axons at longer durations (greater than 1 ms) dendrites are more excitable, and this is the reason that the two curves converge as depicted in Fig 3C.

### Measuring isotropy—threshold amplitude and stimulus duration vs. angle

One dimensional cultures are directed and anisotropic, and can be viewed as an *in-vitro* equivalent to nerve fibers. In the previous section disconnected cultures were studied under stimulation parallel and perpendicular to the orientation of the culture. In continuation, here two different experimental approaches have been used to assess the detailed angular dependence of connected networks. In the first approach we used a fixed high field (0.9 V/mm) and determined the minimal duration for successful excitation. In the second we kept the duration fixed to the value sufficient to stimulate with the field oriented at 90° to the pattern and determined the minimal amplitude required at all other angles. These cultures are relatively young (DIV = 17±3, n = 12) and so their axons are shorter and longer durations are needed for their excitation. But since these are connected network, the most sensitive neurons will stimulate the entire culture.

Fields with a varying angle with respect to the 1D pattern were produced by a double pair of electrodes, (see Fig 1F). By changing the ratio of amplitudes between the two square voltage pulses with the same duration on each electrode the angle of the applied electric field changed without need to physically rotate the culture or the electrode set-up.

**Fig 4:**
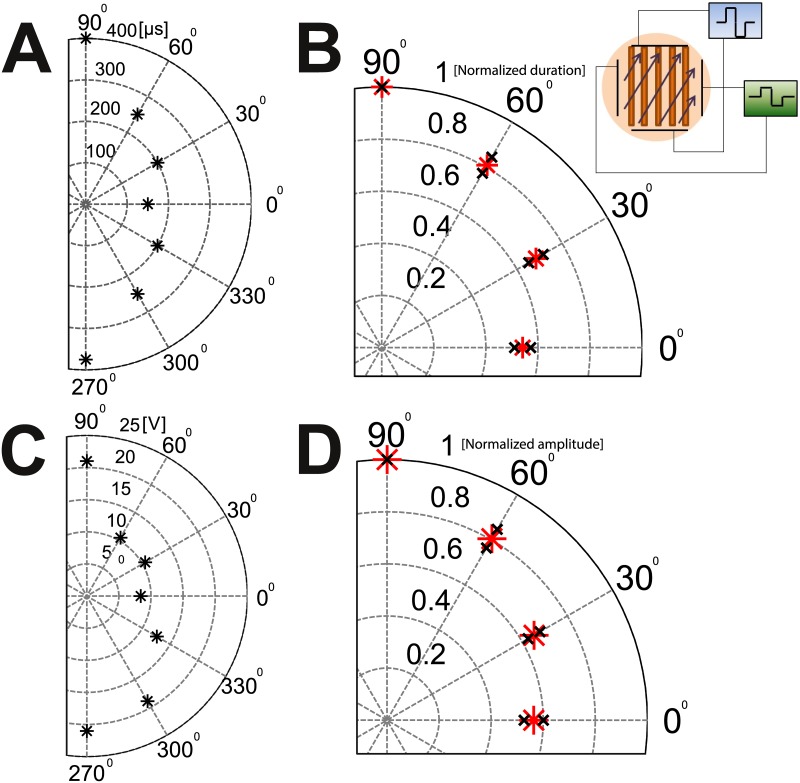
Angular dependence of minimal durations and amplitudes needed for exciting a connected 1D culture with varying angles with respect to the linear culture. (A) A typical example of an experiment with constant amplitude (±22 V) and varying pulse durations. The pulse duration is represented by the distance from the center of the circle. In this example a square pulse of 400 μs was needed to excite with the field perpendicular to the lines (90°), while only 150 μs was needed for excitation parallel to the lines (0°). (B) Fixed amplitude results averaged over 15 different cultures. To allow averaging, the pulse duration in each experiment is normalized by the duration needed to excite the culture in 90 degrees and is plotted as the distance from the center. The red star represents the average, and the black crosses the standard error. The angles are “folded” to the first quadrant (e.g. 330°→30°). (C) A typical example of an experiment with pulse duration held fixed while the amplitude is varied. The distance from the center of the circle is now the amplitude of the square pulse (in Volts) needed for excitation of the culture. The duration was held constant throughout each experiment and was determined as the minimal duration needed for the excitation at ±22 V for 90° orientation. (D) Average over 5 different cultures. The red star represents the average, and the black crosses the standard error. The amplitude of each experiment is normalized by the amplitude needed for excitation at 90°. Inset: Schematic for the setup with 1D networks. The 1D culture is grown on thin lines (width 170 μm, length 11 mm). An external voltage is applied by two perpendicular pairs of bath electrodes, driven by two separate power supplies. The ratio between the amplitude of each pair of electrodes determines the angle of the electrical field allowing measurement of different angles without the need to manually rotate the culture.

To measure changes in pulse duration, the amplitude of the electric field was fixed to 0.9 V/mm and the duration of the square pulse was varied. The minimal duration needed for excitation of the culture was then measured as a function of the angle. Fig 4A presents this minimal duration for a typical 1D culture in the range -90° to 90°. An angle of 0° indicates that the field was parallel to the line (and therefore to the axons), while an angle of 90° indicates that the field was perpendicular to it. For this particular sample, when the field was aligned with the line a 150 μs pulse was sufficient to stimulate the culture vs. 400 μs at the perpendicular orientation.

Fig 4B presents the average over n = 15 experiments after normalizing for each culture to the largest duration needed (perpendicular orientation) and folding the results to the first quadrant. The average duration at 0° was 261±60 μs, while at 90° it was 442±80 μs (p value = 0.024). Averaging over the ratios for the normalized and folded durations gives an averaged ratio of 0.54±0.03. The pulse duration needed for excitation of the dendrites in a connected network is thus about twice that of the axons. The durations measured depend on the length of the axons, which can in turn be influenced by the age of the cultures. Similarly, the amplitude response in axons and dendrites was measured by fixing the pulse duration. Once the minimal pulse duration that produced stimulation for stimulus strength of 0.9 V/mm was determined at an angle of 90° (hardest to stimulate), this pulse width was kept fixed while the stimulus orientation was changed. The electrode setup of Fig 1F was used to stimulate the culture at different angles and different field amplitudes. The minimal amplitude required for excitation was obtained in intervals of 30° for 0° to 90° for n = 5 experiments. The average amplitude needed for stimulation at 0° was 11.7±1.5 V, while at 90° it was 20.8±0.2 V (p value = 0.0003). The results normalized for the largest amplitude (90°) are presented for a typical culture in Fig 4C. Fig 4D presents the average over n = 5 experiments. The average ratio between the amplitude needed for excitation at 0° and the amplitude needed for excitation at 90° was 0.56±0.04. Similar to the results for the pulse duration, at short durations the amplitude needed for excitation of dendrites in a connected network is about twice that of the axons. This also fits well with the disconnected 1D experiment for short durations, where we needed approximately twice the amplitude in order to excite in the perpendicular direction compared with the parallel one.

In contrast, in 2D cultures there were no orientation preference and it responded isotropically, i.e. both the minimal stimulation amplitude and the duration were angle independent (see S7 Text). This lack of orientation dependence can be ascribed the isotropic distribution of axon directions, which are typically oriented in random manner in 2D cultures.

### Simulations—The presence of a leaky end and dendritic integration explain the slower response of dendrites

When a neuron is stimulated by a uniform extracellular field the membrane potential changes differently in different parts of the neuron. The local time course of membrane charging is in part determined by the passive properties throughout the cell, i.e. the specific membrane capacitance and resistance and the resistance of the cytoplasm. The morphology of the neuron also plays an important role in determining the magnitude and time-course of membrane charging. We used passive neuron models to elucidate how these parameters act together to shape the subthreshold response in the different compartments of the neuron and to clarify the contribution of dendrites in this response. The sub-threshold voltage response can also be related to the chronaxies observed experimentally (see Methods). Despite the power of this description very few previous studies (e.g. [41]) have concentrated on the passive response.

In the SI section S8 Text, we characterize the passive response of the typical structures in the cell (dendrite, soma, and axon), in isolation from the rest of the neuron. This clarifies which parameters influence the chronaxie and which chronaxie values can be expected for dendritic structures. The parameters of the neurite that have a strong effect on the chronaxie are the diameter, the intracellular resistance R_i_, and the length of the cable L, for details see S9 Text. We show there that the time response of dendrites is on the order of a few hundred microseconds, while that of axons is much longer in the passive regime. The introduction of asymmetry, arising from a more complicated structure of axon-soma-dendrite, combined with the typical dendrite diameters, prolongs the dendritic response.

**Fig 5:**
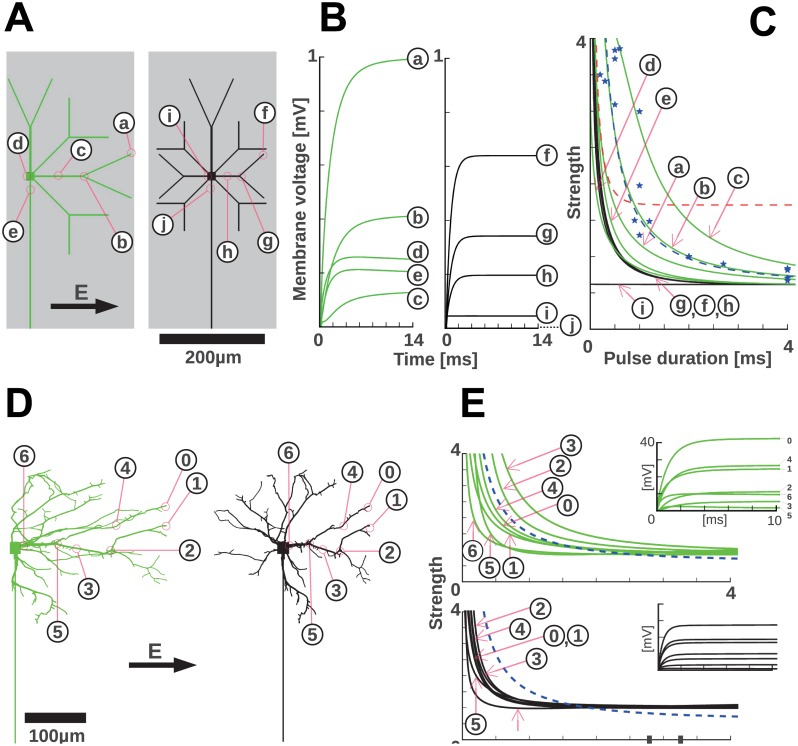
Simulations show that complex morphology and asymmetry dominate the charging kinetics in short neurites. (A) Model neurons featuring an extended dendritic tree that spreads out to cover the width of the structured substrate in the patterned cell cultures used in this study. Axons follow the direction of the strips (grey area) that denote the experimental line on which the neurons grow. The indicated positions within the dendritic tree are the locations for which charging kinetics and derived, normalized strength-duration plots are shown in B and C. If the soma is positioned at the center of a substrate strip, the dendritic tree can be left-right symmetric (black). For off-center locations, the arborization is asymmetric (green). (B) The charging kinetics for the models in A are shown. Symmetric dendritic trees lead to a faster charging kinetics: the soma is not charged and therefore no current flows into the axon. In the asymmetric case, longer dendrites occur (A left) and this favors slower charging kinetics. In addition, the soma is polarized and the resulting current flux into the very long axon contributes another slow timescale of charging (see panel G of S4 Fig). In A and B the tip segments a and f are the most depolarized, but their chronaxies do not match the experimentally observed ones. This could indicate that tip segments are not efficient in causing excitation of the cell. (C) From the charging curves in B normalized strength-duration curves are computed (see Materials and Methods). Corresponding to the uniformly fast charging of symmetric dendritic trees, the strength-duration curves are almost uniform throughout the arbor. Asymmetric arborization creates larger chronaxies. Blue stars and the dashed blue line represents the experimental data and fit for dendritic stimulation (i.e. perpendicular to lines) and the dashed red line the axonal stimulation fit for comparison (Fig 4). (D) A representative reconstructed cultured neuron (black, obtained from neuromorpho.org, Id: NMO_07978, from the dendritic arborization studies in primary cultures [52], and an asymmetric version (green) are used to obtain realistic charging curves for realistic dendritic trees. (E) The charging kinetics (insets) and the derived, normalized strength-duration curves for the reconstructed neuron. These confirm that in the absence of a pronounced lateralization (black) chronaxies are smaller than for pronounced left-right asymmetry (green). The chronaxie expected for dendritic stimulation of a realistic, asymmetric arbor falls in the range of observed chronaxies (dashed blue line). Symmetric dendritic trees would cause significantly shorter chronaxies.

In the following we utilize the results obtained in the SI and turn to more complex structures of artificial neurons and reconstructed cells. We show that, although unexpected from the properties of individual cables, the experimentally observed dendritic chronaxie indeed corresponds to the sub-threshold response of certain dendritic arbors. The effect of the asymmetry extends to tree-like structures and helps identify a plausible place for the origin of the dendritic response. In Fig 5C and 5D, the response of artificial trees, asymmetric or symmetric, to a field perpendicular to the axon are compared. The responses in different spots of the arbors show that, throughout the dendritic tree, the membrane voltage responds slower in the asymmetric than in the symmetric case. Only at locations around the soma the asymmetric and the symmetric case display similar time constants. Consequently, the normalized strength-duration responses (Fig 5E) feature larger chronaxies for the asymmetric case, covering a range of 375 μs to 2.4 ms compared to the very homogeneous kinetics in the symmetric tree with chronaxies around 400 μs. While cell morphologies are never perfectly symmetric, the stimulation of the reconstructed tree of a typical hippocampal neuron in a standard, unpatterned primary culture essentially confounds the conclusions from the symmetric model (black in Fig 5D and 5E).

In the quasi-1D culture, that we used in this study, the dendrites of somata, located anywhere on a 200 μm wide line, can only grow within the limits of this line. Hence, a large fraction of the dendritic trees will be asymmetric with respect to the direction perpendicular to the stripe. To simulate this, we modified the reconstructed arbor to create an asymmetric structure with the soma at one extreme. As expected, the longer maximum length of the dendrites, together with the effects of asymmetry gives rise to larger chronaxies, on the order of 375 μs to 2.4 ms. In comparison, the soma has a faster effective response. This is an effect of its larger diameter as seen in the d = 5 μm dendrite in panel A in S4 Fig.

Regions proximal to the soma display a combined response (see for instance case (5) in the asymmetric case). Although the soma seems to have a much faster response we doubt the amplitude for the stimulus at this point (17% of the tip response, not shown) is enough to initiate a response faster than at the dendrites.

The voltage response of the axon initial segment is comparable to the response at the soma (Fig 5C). We believe that similarly as the soma, the amplitude in this case is not enough to initiate a response. In addition, if the axon initial segment was involved in the response of the dendrites then their response would probably be as fast as the axonal response. The only time constants that correspond to the observed responses are those of the branching points of the dendritic tree indicating a dendritic spike initiation.

### Simulation—Chronaxie of Axons

The passive model that we employed thus explains the chronaxie of the dendrites, which is considerably shorter than the membrane time constants, as resulting from a combination of geometry and asymmetry. For the axons, however, we were not able to reproduce the experimental chronaxie, and the passive equations consistently gave times that are on the order of several milliseconds (data not shown). We propose, therefore, that *active* processes involving channels and their relative densities and dynamics should be implicated. For example, sodium channel inactivation dynamics in the axons can significantly change its chronaxie. As we show below, a high density of A-type potassium channels in the dendrites reduces their chronaxie and brings it close to the chronaxie that the axons exhibit in the experiment. It is possible that other channels which are open near the resting membrane potential also play a role.

### Active properties affect the fast response of axons

A-type channels are known to be important in controlling and reducing excitability in the dendrites. To examine how reducing active processes in the dendrites affects the activation of the neurons, fast (A-type) potassium channels were blocked by 4-AP. We compared a fixed directed field to a rotating field excitation in 2D connected cultures under blockade of A-type potassium channels. Since very few axons will be oriented in a particular single direction, the uni-directional field excites mostly dendrites. We therefore expect that the duration of the uni-directional electric field applied will have to be long to achieve stimulation of the culture. In contrast we expect that for the rotating electric field pulse the excitation will be axonal since the electric field does spend a small amount of time at each angle. Thus the rotation “collects” or integrates all possible directions, and can be expected to excite the most excitable axons growing in all the scanned directions.

**Fig 6:**
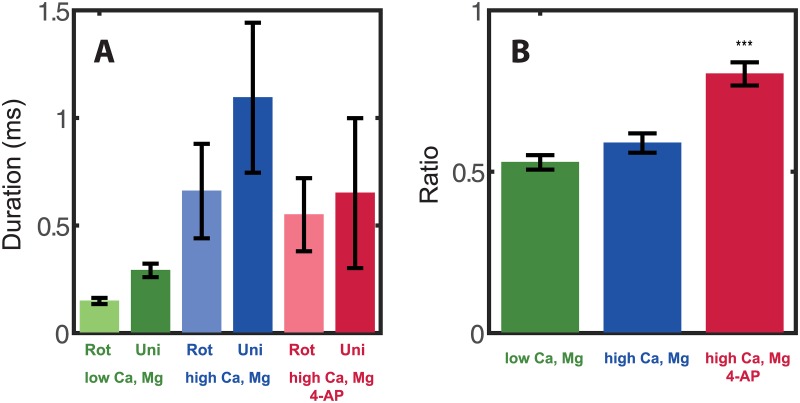
Comparison of excitation with a rotating vs. uni-directional field with and without the A-type channel blocker 4-AP. (A) Pulse durations needed for excitation with rotating (light green) vs. uni-direction (dark green) for [Ca2+] = 1 mM and [Mg2+] = 1 mM, similarly for [Ca2+] = 4 mM and [Mg2+] = 2 mM (center, light and dark blue) and for [Ca2+] = 4 mM and [Mg2+] = 2 mM with addition of 2 mM 4-AP (right, light and dark red). The effect of the change in concentration of ions is to decrease the membrane voltage, countering the increased excitability caused by blocking the A-type channels, which causes the durations needed to be overall about four times longer with higher ionic concentrations. (B) The ratios between durations needed for excitation with a rotating pulse and a uni-directional pulse for the three conditions measured: low concentrations of [Ca2+] and [Mg2+] (green), high concentration of [Ca2+] and [Mg2+] (blue), and high ionic concentrations with the addition of 4-AP (red). Addition of calcium and magnesium does not affect the ratio. Addition of 4-AP does shorten the duration of the uni-directional field but not of the rotating pulse.

As explained in the Methods section, the measurements taken with 4-AP are performed at elevated ionic concentrations of [Mg^2+^] and [Ca^2+^], which stabilize the activity of the neurons once A-type channels are blocked. The baseline was measured without 4-AP, first at normal ionic concentration (Fig 6A and 6B, green), and then at elevated [Mg^2+^] and [Ca^2+^] (Fig 6A and 6B, blue). We first measured the minimal pulse duration needed for excitation of a 2D connected network of a rotating electrical field at the maximum available amplitude of 22 V. This was compared to the minimal pulse duration needed for excitation of the same culture with a square uni-directional pulse. The mean durations with a rotating field and with a uni-directional square pulse are depicted in (Fig 6A). As can be seen from Fig 6A, longer durations (~4 fold) were needed with elevated levels of [Mg^2+^] and [Ca^2+^] (blue bars compared to green bars). However, the ratio of the durations of a rotating pulse vs. those with a uni-directional pulse did not change significantly (p value = 0.14) with the change in ionic concentrations: At regular ionic concentrations it was 0.53±0.02 (n = 23, Fig 6B, green). After addition of [Mg^2+^] and [Ca^2+^], the ratio was 0.59±0.03 (Fig 6B, blue), statistically indistinguishable from the value measured at the lower ionic concentrations. The two values measured for the ratios are notably similar to the results given above for parallel versus perpendicular excitation in the 1D connected networks. In summary, for our control baseline, both at normal and at elevated [Ca^2+^] and [Mg^2+^], almost twice the pulse duration was needed in order to excite 2D cultures using uni-directional electrical field compared to a rotating field.

We then applied 4-AP to the cultures. At 2 mM concentration 4-AP is a selective blocker of the A-type potassium channels, which are more abundant in dendrites. It should therefore have a stronger influence on dendrites, changing their active properties. When applying 4-AP the duration for the rotating pulse (axonal excitation), the duration needed for excitation stayed about the same, but the duration of the uni-directional pulse (dendritic excitation) was reduced (Fig 6A, red). The ratio of the rotating pulse duration to the square pulse duration was changed to 0.8±0.04 (Fig 6B, red), averaged over n = 10 cultures (compare to 0.59±0.03 for the no-4-AP case, p-value = 2e-4). This confirms our hypothesis that the active contribution of the A-type channels to the process of excitation is considerable in the dendrites, but less so in the axons.

## Discussion

The mechanism by which macro-stimulation activates neurons is important both conceptually and for applications such as DBS and TMS. It is therefore important to determine how different parts of the neuron respond to different amplitudes and durations of external fields during macro stimulation. Our success in elucidating aspects of this question relies on the technically innovative methodology that we have employed. First of all, we used population statistics of cells to determine average axon and dendrite responses. This gives us a good representation of neurons in the hippocampus and eliminates noise since single neurons are susceptible to artifacts of structure and environment. This is enabled by the second advance which consists of using cultures of neurons that are grown on patterned substrates, orienting the axons of the whole population along the same direction. Pharmacological treatment then allows for a separation of the neuronal network into the individual responses, whose statistics are combined for a reliable and reproducible measurement.

The directionality of the axons in the culture thus allows for the separation of the dendritic and axonal responses by orienting the electrical field orthogonal or parallel to the pattern. Axons and dendrites were found to have significantly different chronaxies and rheobases. The chronaxie of axons is on the order 110 μs, while for dendrites it is 0.9 ms. Furthermore, the rheobase of axons is 2.3 larger than that of dendrites. This means that with pulses of short durations, axons are more easily excited than dendrites. Perhaps the most counter-intuitive result is that at long durations (> 1 ms) dendrites are more excitable, supporting neuronal excitations with stimulus intensities that are less than half of those required for axonal stimulation. This has obvious consequences for TMS and DBS, whose electric field directionality is fixed and thus must be oriented along the axons, or else have durations that can excite dendrites. Current commercial technology limits magnetic stimulation to durations less than 1 ms by technical considerations, while DBS excitation can easily be prolonged beyond the millisecond range.

One limitation of our experiment is that the ideal differentiation would be between excitation by dendrites alone versus excitation by axons alone. However, with the field parallel to the one-dimensional culture our setup does not distinguish between the concurrent contributions of the dendrites and the axons. Similarly in the two-dimensional cultures without rotation, there are some axons that will be aligned with the direction of the field, although this number will typically be small. Ideally, one would like to design a system where the axonal contribution may be singled out. Another limitation of our present study is that the active processes that we investigated were limited to the effect of A-type channels on dendrites. The use of 4-AP necessitated changing the medium conditions to reduce the firing rate, and the overall result can only be used as an indication that active processes involving the dynamics of ion channels are an important factor in determining the firing threshold. This is in essence counter to the theoretical description of the strength-duration curve in terms of the passive cable equation involving resistance and capacitance. This ties in to limitations in the theoretical model, which relies on the passive membrane and does not take into account the contribution of ion channel dynamics. The inability to describe the short time scales of the axon may relate to this limitation.

Chronaxie measurements were conducted with disconnected networks, where precise measurements and population statistics could be carried out, but connected networks are also obviously of interest, particularly in the context of living organisms. Our measurements with connected networks were conducted with much younger cultures than the measurements with disconnected networks. Younger cultures have shorter axons, thus the typical durations need to be larger. This is balanced by the fact that when stimulating a connected network it suffices to excite the most sensitive neurons (3 to 5 percent of the network) in order to propagate activity to the rest of the network [42, 43]. The ratio of amplitudes needed for stimulation at short durations of connected networks at 0 degrees to 90 degrees (Fig 4D) is very similar to the ratio of amplitudes needed for stimulation of disconnected networks (Fig 3) at the same orientations (a ratio of about ~0.6).

In the brain, excitation by a uniform electric field is most efficient in axons that are bent [44, 45]. In our experiments the electric field created by the parallel pair for bath electrodes is highly uniform (see S2 Fig). Such a uniform field does not create a potential difference in an infinitely extended conducting cable; it will rather move charges and cause a current. To produce a potential difference charges have to accumulate, and that occurs if the neurite bends or bifurcates, or where it terminates. Charge will accumulate at the end of a cable like a on a capacitor, having a long effective length for the axon. This is in fact a special feature of the one dimensional patterns, in which the axons do not bend, and their total (finite!) length contributes to the creation of a potential. In contrast, in the two dimensional case the potential created depends strongly on the bends, as well as on the projected length of the axon in the direction of the electric field. Most axons will not be completely straight or parallel to the direction of the electric field, and charges will accumulate at the bends. The total potential difference that will depolarize the bent axon will therefore be small, and very few neurons will cross the threshold. Since a minimal number of neurons is needed to excite the whole network [42] it is usually harder to excite neurons in two dimensional cultures with short pulses.

Our results show that dendrites and axons respond in a very different time-scale, almost an order of magnitude difference. This is somewhat surprising, since a-priori all parts of the neuron have similar passive membrane properties, and should therefore respond with the same time constant. According to the naïve, passive cable equation we would expect that the much longer axon will depolarize to a higher value and with a longer time constant than the dendrite. Our experimental measurements indicate the opposite: axons have a shorter chronaxie, and a higher rheobase. Our analysis and simulation give a first indication for the cause of the discrepancy in that the resistance that contributes the most to the chronaxie is the internal resistance rather than the membrane resistance. This is in contrast to simple models such as those suggested by Lapicque, in which charging the membrane does not take into account the need for the current to pass through the neurite along the axial axis. It fits better models in which the electrotonic length scale is used to modify the relevant time scales. When the spatial asymmetric distribution of the dendritic tree of the neuron is taken into account, dendritic chronaxie increases to the orders we measured in the experiment. This is a direct result of leaky boundary conditions at the end of the neurite that is connected to the soma.

In this regard the results of Fig 5 should be taken to show that dendritic time scales can be in the range of the measured chronaxies. Of all the different segments of the cell described in Fig 5C, the bifurcation geometry has the time constant that is closest to the observed one. In a more realistic structure such as in Fig 5D, the shortest chronaxies occur at the bifurcations (segments 5 and 6). That might indicate that in the real neurons, excitation at the very tips does not occur, and other segments play the dominant role in initiating the action potential. Indeed, multi-compartment models constructed to match experimentally observed dendritic impedances show that single branch action potentials often fail to invade the larger parent branch due to impedance mismatches [46]. A systematic study of the relation of AP propagation and dendritic tree morphology also shows the possibility of AP transmission failure at branching points [47]. Therefore, it is plausible that depolarization at branching points, which naturally recruits parent and children branches, faces no impedance mismatch problem and can thereby successfully excite dendrites and subsequently the axo-somatic compartment.

The short chronaxies we obtained for the axons, on the order of 100 microseconds, are more characteristic of myelinated neurons, where a whole range of chronaxies has been observed, depending on the axon geometry and degree of myelination [48] At the slowest extreme, the chronaxies are on the order of a millisecond, which agree with our simulations for short, passive and unmyelinated neurites and with the measurement for the dendritic chronaxie. Similarly, [19] reviewed experimental results of a few types of myelinated and non-myelinatd axons, which fall in the range of 40–600 microseconds and 400–6000 microseconds respectively. Specifically, pyramidal tract neurons were measured to have 100–450 microseconds chronaxies, and rat hippocampus 240–460 microseconds. These were taken from adult animals, which means that the axons are already myelinated, but as it was shown in [49] this is a thin myelination. This means that our results are quite similar to previous reported chronaxies measured in live animals. The cable equation gives a much more satisfactory agreement in myelinated neurons with experiments [37, 50]. However, the possibility of contribution from active processes such as sodium deactivation, has been raised in that context previously.

An intriguing possible cause for the longer chronaxie of dendrites in comparison of axons is indeed the existence of active processes in the membrane that affect excitability. One possibility is that channel activation modifies the resistivity of the membrane and therefore also their time constant. It is usual to assume that sodium channels dominates such effects, but the role of potassium channels is also important [51]. In fact [51] have modeled the threshold kinetics directly, and in their model the excitation threshold indeed increases with the excitation duration due to active processes. A second possibility is that the threshold for activation of an action potential is fundamentally different depending on whether it is excited in an axon or in a dendrite, created by variability in the distribution of ion channels in the membrane. As an example, we have shown that blocking the fast response of the A-type potassium channels with 4-AP causes an increase in overall excitability, and brings the dendritic excitability closer to that of the axons. This most likely correlates with a corresponding change in the chronaxies, although it does not explain the discrepancy with the passive cable equation. In summary, we are not able at present to discern whether the measured differences in time scales are explained in terms of structural factors such as geometry and asymmetry or whether active factors are involved. Our belief is that in fact a combination of active and passive mechanisms is in play.

## Supporting Information

S1 Fig Typical cultures growing in 2D and 1D patterning. (A,B,C) Images of one dimensional culture at 2, 6 and 11 DIV respectively. When the culture is still young (A) some of the neurons cluster outside the pattern, but as the network matures more and more neurons migrate towards the pattern (B), until almost all of them form a well-connected neuronal network which is restricted to the pattern itself (C). In C, the fully mature network has no connections outside the pattern. (D) Neu-N (neuron specific nuclear protein) staining of a 1D culture. White lines are a guide to the eye, delineating the borders of the imposed linear pattern for growth of the neurons. (E) An image of a 2D neuronal culture. (F) Fluorescence microscope image of a Tau-GFP transfected culture. Only about 1% of the cells are transfected. Panels D,F courtesy of Prof. Ofer Feinerman, Weizmann Institute.
(EPS)

S2 Fig Controlling for the homogeneity of the field produced by the pair of parallel platinum bath electrodes. The potential produced within the recoding medium as measured by a needle probe electrode, which was successively moved in the sample cell at a resolution of 1 mm x 1 mm grid. The fields are homogenous up to about 10%, with the largest deviations at the boundary that is parallel and close to the bath electrodes. Results are shown for pulse duration of (A) 0.1 millisecond, (B) 1 millisecond and (C) 4 milliseconds.
(EPS)

S3 Fig Connected two dimensional cultures are isotropic and have no preferred orientation for excitation. (A) The duration (in μs) of the minimal square pulse needed for excitation, measured by changing the angle between the sample and the electrodes in steps of 15°. The distance from the center of the circle represents the pulse duration. Different colors represent different cultures (total of seven cultures). (B) The minimal duration needed for excitation was normalized in amplitude and shifted in angle after spherical harmonics decomposition (see S7 Text). The distance from the center of the circle denotes the normalized duration. Inset: Schematic of the basic setup. A pair of bath electrodes is inserted into a dish with recording media. An external voltage is applied by a signal generator with the shape of a square pulse with 50% duty cycle and amplitude of ±22 V. The electrodes are positioned above the culture at a height of 1 mm. The sample was rotated manually with respect to the electrodes to vary the orientation of the electric field.
(EPS)

S4 Fig Simulations show that neuronal morphology impacts the kinetics of membrane voltage excursion under extracellular macro-stimulation. Simplified neuron models are schematically shown, together with diagrams of the change in the membrane voltage induced by an extracellular field (150 V/m) across the neuron and over time. Field orientation is indicated by arrows, the field was switched on after 200 μs. (A) Cables with a length L, on the order of the electrotonic length constant (L≈λ), charge up with a time constant of about τ_m_/(1+ π^2^λ^2^/L^2^) (see Text). (B) Shorter (200 μm) neurites, e.g. dendrites, charge up much faster, with the cell body charging in as little as 1 μs but only by about 2 mV. When soma and dendrite are connected, the charging kinetics changes slightly, as a second, slower phase is appearing. Most prominently, the symmetry between the voltages at the opposite ends is broken by much more than the depolarization of the isolated soma. (C) When the long neurite from A and the short neurite from B are connected via a soma the spatial and temporal patterns mix. In this case the amplitude of the field was reduced to produce a maximum of 1mV depolarization. The fast and slow timescales are separately resolved in (D, E) (a), (b) denote neurite thickness, as depicted in C (a = red, b = blue). While the charging of the distal axon ending is changed only slightly (axon (b,a) and red curve in A), the proximal part shows a clearly biphasic charging (dendrite (a,b)). For a wider dendrite (5 μm diameter (a)) even the sign of the somatic membrane potential changes during the process (green soma (a)). (F) Influence of dendrite length, diameter and passive properties, axial resistance and membrane resistance on the membrane charging. Note the logarithmic timescale. In the tip of a single, isolated dendrite, the final depolarization amplitude is crucially determined by the dendrite length. For shorter dendrites the depolarization is smaller, and the charging kinetics speeds up (orange, purple, blue). Reduction of the effective axial resistance, either by an increase of the diameter (blue to green) or by a decrease of the specific axial resistance R_i_ (brown to blue) accelerates the charging, although these parameters have no influence on the membrane time constant τ_m_. In contrast, a 10 fold decrease of the specific membrane resistance R_m_, which leads to a proportional change in τ_m_, has almost no influence on the kinetics and very little influence on the amplitude of membrane charging (black and blue). (G) The addition of an axon perpendicular to the field direction introduces a longer timescale to the charging process. The membrane voltage in the dendritic tip is increasing considerably (green and orange).
(EPS)

S1 Text Preparation of primary cultures. (DOCX)

S2 Text Calcium sensitive imaging. (DOCX)

S3 Text Electric Stimulation. (DOCX)

S4 Text 4-AP experiments. (DOCX)

S5 Text Network disconnection for the strength. (DOCX)

S6 Text Extracting the chronaxies and parameters from the cable simulations. (DOCX)

S7 Text 2D connected cultures respond isotropically. (DOCX)

S8 Text Simulations—Time course of membrane. (DOCX)

S9 Text Simulations—Axial resistance and diameter. (DOCX)
