# Supplementary material for: Chronaxie Measurements in Patterned Neuronal Cultures from Rat Hippocampus
Source: PLoS One. 2015 Jul 17;10(7):e0132577. doi: 10.1371/journal.pone.0132577 (PMC4506053; doi:10.1371/journal.pone.0132577)
Supplement: S1 Text — (DOCX) [file pone.0132577.s005.docx]

## Preparation of primary cultures

Pregnant rats were anesthetized with veterinary pentothal at a dose of 0.5 ml/kg to efficiently anesthetize the animal while minimizing the risk of affecting the embryo brains by the barbiturate. This was followed by cervical dislocation and swift extraction, which prevents damage to the brain tissue. All procedures were approved by the Weizmann Institutional Animal Care and Use Committee. The dissection was performed following [[1](#_ENREF_1)].

Two-dimensional (2D) and semi one-dimensional (1D) cultures were used in the experimental procedures. The 2D cultures were grown on circular 13 mm glass coverslips (#0,1 Menzel-Glaser, Germany). The 1D cultures were grown on patterned circular 13 mm glass coverslips, with only 5 strips (0.170 mm wide and 11 mm long) available for cell adhesion [[2](#_ENREF_2)]. Cells were plated at a density of 650,000 cells (1D cultures), and of 850,000 cells (2D cultures) per coverslip. The cells attach to the lines only, One-third to one-half of the medium volume was replaced every day starting from day 9 in culture.

The 1D patterns are prepared in two steps. The entire coverslip is first coated to make it inert to protein and thus to cell adhesion, and then lines are scratched through this coating and the entire coverslip is immersed in a solution of adhesion proteins. The inert coat is composed of 4 layers, with an initial deposition onto the glass coverslips of an 8 Å of chromium followed by a 40 Å layer of gold, and then immersion in octadecanthiol. The molecules of octadecanthiol are linear and highly hydrophobic with a high affinity for gold on one end, causing them to self-assemble into a hydrophopbic monolayer. For the fourth layer the coverslip is immersed in a solution of Pluronics F108 Prill. Lines are then etched into the desired pattern with an HP plotter. Finally the coverslip with the pattern etched on it is put in a solution of laminin and fibronectin to allow cell adhesion to the pattern. An image of 1D cultures at different DIVs can be seen in Fig. S1. The neurons are initially distributed homogenously, but as the culture matures they are repelled from the coverslip towards the 1D pattern.

1. Feinerman O, Segal M, Moses E. Signal propagation along unidimensional neuronal networks. J Neurophysiol. 2005;94(5):3406-16. Epub 2005/07/29. doi: 00264.2005 [pii]

10.1152/jn.00264.2005. PubMed PMID: 16049148.

2. Feinerman O, Rotem A, Moses E. Reliable neuronal logic devices from patterned hippocampal cultures. nature physics. 2008;4(12):967-73. PubMed PMID: ISI:000261386000021.
