# Supplementary material for: Chronaxie Measurements in Patterned Neuronal Cultures from Rat Hippocampus
Source: PLoS One. 2015 Jul 17;10(7):e0132577. doi: 10.1371/journal.pone.0132577 (PMC4506053; doi:10.1371/journal.pone.0132577)
Supplement: S2 Text — (DOCX) [file pone.0132577.s006.docx]

## Calcium sensitive imaging

CI has three distinct advantages of other measuring methods when using electric fields. First, CI avoids induced interference in the recordings since optical measurements are unaffected by the electric field. Second, imaging provides simultaneous monitoring of large populations of cells. This is more efficient than searching for single neurons that respond to the stimulation. Finally, in comparison to voltage-sensitive dyes CI has a large signal to noise ratio.

The major shortcoming of calcium imaging is the long time (order of a second) that it takes the fluorescence to recover. In our experiments this is not critical because of delay between network bursts is typically longer than one second. What interests us is the response of the network to stimulation, and for that the much shorter rise time of the calcium-sensitive fluorescence (order of milliseconds) is the relevant parameter.

To image calcium transient cultures aged between 10 and 40 days were incubated for 60 minutes in the recording solution (128 mM NaCl, 4 mM KCl, 1 mM CaCl_2_, 1 mM MgCl_2_, 45 mM sucrose, 10 mM glucose, and 10 mM HEPES; pH is titrated to 7.4) in the presence of 4 μg/ml cell-permeant Fluo4-AM (Invitrogen, Carlsbad, CA) dye. Cultures were then placed in fresh recording solution and imaged on an Axiovert 135TV inverted microscope (Zeiss, Oberkochen, Germany). The calcium transients were imaged at 50 Hz following the same video capture procedures explained in [[1](#_ENREF_1)] while electric or magnetic pulses were applied.

1. Rotem A, Moses E (2008) Magnetic stimulation of one-dimensional neuronal cultures. Biophysical Journal 94: 5065-5078.
