# Supplementary material for: Chronaxie Measurements in Patterned Neuronal Cultures from Rat Hippocampus
Source: PLoS One. 2015 Jul 17;10(7):e0132577. doi: 10.1371/journal.pone.0132577 (PMC4506053; doi:10.1371/journal.pone.0132577)
Supplement: S3 Text — (DOCX) [file pone.0132577.s007.docx]

## Electric Stimulation

To produce independent signals in each of the electrodes two signal generators (BKPrecision 4079, Keithley 3390) were used, with no common ground so they were completely decoupled. The signal generators were connected to homemade amplifiers which in turn fed the platinum wires.

Electric stimulation was given in one of a few configurations:

1. Single pair electrodes induce a uniform electric field with a constant amplitude and orientation. This was applied for both 1D and 2D cultures (Fig 1D). The signal fed through the signal generators is a single pulse of a varying duration and amplitude square pulse with 50% duty cycle (Fig 1A).
2. Two pairs of electrodes (with no common ground) with a different amplitude in each, allows producing a uniform electrical field with varying amplitudes and angles with respect to the culture. This was applied both to 1D and 2D cultures (Fig 1E and 1F). The signal fed through each signal generators is a single pulse of a varying duration and amplitude square pulse with 50% duty cycle (Fig 1A).
3. Two pairs of electrodes (with no common ground), where the inputs are a single cycle cosine and sine wave creating a constant amplitude rotating electrical field. This was used to stimulate 2D cultures (Fig 1G).

The amplifiers were fed with two Matrix MPS-3005 LK-3 power supplies. Changing the relative amplitudes in the two pairs of electrodes, compared to each other, changes the orientation of the electric field.

To control for the uniformity of the induced electric field a special probe electrode was inserted into the conductive bath with culture dish installed, and the electric potential was measured at a height of about 1mm above the glass coverslip, with a resolution of 1mm in both directions. The electric field was typically homogeneous to within 10% in space, and at worst to about 20%, see S2 Fig.
