# Supplementary material for: Chronaxie Measurements in Patterned Neuronal Cultures from Rat Hippocampus
Source: PLoS One. 2015 Jul 17;10(7):e0132577. doi: 10.1371/journal.pone.0132577 (PMC4506053; doi:10.1371/journal.pone.0132577)
Supplement: S4 Text — (DOCX) [file pone.0132577.s008.docx]

## 4-AP experiments (connected two dimensional networks)

Application of 2 mM of 4-AP resulted in the technical difficulty that the culture became highly excitable, resulting in an almost continuous spontaneous activity that practically eliminated the possibility of externally exciting the culture. To reduce the spontaneous activity, 4 mM CaCl_2_ and 2 mM MgCl_2_ were added to the recording solution instead of 1 mM CaCl_2_ and 1 mM MgCl_2_. In this way we obtained quiescent periods when we could apply the external excitation without the spontaneous activity interfering. Results were taken with the new concentration of Mg and Ca twice: without 4-AP and then with 2 mM 4-AP, and the results presented are always a ratio of these two values.
