# Supplementary material for: Chronaxie Measurements in Patterned Neuronal Cultures from Rat Hippocampus
Source: PLoS One. 2015 Jul 17;10(7):e0132577. doi: 10.1371/journal.pone.0132577 (PMC4506053; doi:10.1371/journal.pone.0132577)
Supplement: S5 Text — (DOCX) [file pone.0132577.s009.docx]

## Network disconnection for the strength-duration curve (one dimensional networks)

To analyze the network connectivity it is useful to differentiate excitation of activity in neurons that originates by the external electric field from stimulation by neighboring neurons. In order to look only at the direct response to the electric field the synapses to neighboring neurons need to be disconnected so that neurons are only affected by the electric field and not by other neurons firing. Synaptic connections between neurons in our culture are both excitatory and inhibitory in nature, and are dominated by AMPA, NMDA, and GABA receptors. To block synaptic transmission we apply 10 μM of the NMDA receptor antagonist 2-amino-5 phosphonovaleric acid (Sigma-Aldrich), 40 μM GABA receptors antagonist bicuculline-methochloride (Sigma-Aldrich) and 10 μM of the AMPA/kainate receptor antagonist 6-cyano-7-nitroquinoxaline-2,3-dione (CNQX, Sigma-Aldrich). The use of these blockers completely breaks down the network structure and connectivity [[1](#_ENREF_1), [2](#_ENREF_2)] .

1. Breskin I, Soriano J, Moses E, Tlusty T. Percolation in living neural networks. Physical Review Letters. 2006;97(18).

2. Soriano J, Breskin I, Moses E, Tlusty T, editors. Percolation approach to study connectivity in living neural networks2007. Granada, Spain: American Institute of Physics.
