# Supplementary material for: Chronaxie Measurements in Patterned Neuronal Cultures from Rat Hippocampus
Source: PLoS One. 2015 Jul 17;10(7):e0132577. doi: 10.1371/journal.pone.0132577 (PMC4506053; doi:10.1371/journal.pone.0132577)
Supplement: S6 Text — (DOCX) [file pone.0132577.s010.docx]

## Extracting the chronaxies from the cable simulations

The strength duration curve is defined as the set of minimal stimulation intensities that suffice to create a supra-threshold response for the respective stimulus durations, and varies from segment to segment of the cell. However, as explained above, V_rh_ may vary between segments and is in general not accessible numerically. Instead, we introduce a normalization to bring all the SD curves to the same voltage at long times.

Although the rheobase cannot be extracted from the simulation, the chronaxie is independent of the rheobase and can be obtained. For any choice V_rh_ of threshold, the chronaxie is just the time point t_chr_ at which the voltage trajectory reaches half the final depolarization V(t_chr_)=V_rh_/2. The strength duration curves produced in this way are actually the inverse of V(t), normalized to a strength of 1 for a duration of 2 ms. This is similar to the normalization done for the experimental data.

Whether the depolarization of a particular segment will cause excitation of the whole cell will depend in general on many different factors, which are difficult to assess. For example, local excitation of thin processes often fails to propagate, as the amount of current produced in the thin processes is insufficient to depolarize the wider parent branches. In addition, the voltage dependence of active processes underlying local excitation can dictate the local value of V_rh_, and these are largely unknown. Given these factors, it is not obvious which part of the dendritic tree is most excitable, and we therefore concentrate on evaluating only the chronaxie in different neurites, asking whether the value of the chronaxie obtained is similar to the experimentally measured ones.

## *Numerical parameters and geometries for the cable simulations*

The effect of the extracellular fields on the membrane potential was calculated with numerical compartmental models of individual cables, extended synthetic neurons (following Rall’s 3/2 rule) and reconstructed cells. The reconstructed hippocampal culture cell we used was obtained from neuromorpho.org (Id: NMO_07978, rat, 12 days old culture) from the dendritic arborization studies of [[1](#_ENREF_1)]. All the models were passive with a specific membrane resistance of 30 kΩcm² and a specific membrane capacitance of 1 µF/cm². The axial resistivity for dendrites and soma was 200 Ωcm as estimated for dentate gyrus granule cell dendrites [[2](#_ENREF_2)] and CA1 pyramidal neuron dendrites [[3](#_ENREF_3)]. Very few works describe the axial resistivity of the non-myelinated axon, with the best modern estimates obtained from the non-myelinated mossy fiber. The estimate for this fiber is between 50 and 120 Ωcm [[4](#_ENREF_4), [5](#_ENREF_5)]. A resistivity of 100 Ωcm, half of the dendritic axial resistance, was used in the axons belonging to synthetic trees and reconstructions. Some simulations were run with both values 100 and 200 Ωcm for the axial resistance in dendrites and found only small differences in the response at the dendrites (data not shown). The 1 mm neurites representing axons in panel G in S4 Fig used a resistivity of 200 Ωcm to allow for a straightforward comparison with shorter neurites.

1. Peng Y-RR, He S, Marie H, Zeng S-YY, Ma J, Tan Z-JJ, et al. Coordinated changes in dendritic arborization and synaptic strength during neural circuit development. Neuron. 2009;61(1):71-84. doi: 10.1016/j.neuron.2008.11.015.

2. Schmidt-Hieber C, Jonas P, Bischofberger J. Subthreshold Dendritic Signal Processing and Coincidence Detection in Dentate Gyrus Granule Cells. The Journal of Neuroscience. 2007;27(31):8430-41. doi: 10.1523/jneurosci.1787-07.2007.

3. Golding NL, Mickus TJ, Katz Y, Kath WL, Spruston N. Factors mediating powerful voltage attenuation along CA1 pyramidal neuron dendrites. The Journal of Physiology. 2005;568(Pt 1):69-82. doi: 10.1113/jphysiol.2005.086793.

4. Alle H, Geiger JR. Combined analog and action potential coding in hippocampal mossy fibers. Science. 2006;311(5765):1290-3. Epub 2006/03/04. doi: 311/5765/1290 [pii]

10.1126/science.1119055. PubMed PMID: 16513983.

5. Scott R, Ruiz A, Henneberger C, Kullmann DM, Rusakov DA. Analog modulation of mossy fiber transmission is uncoupled from changes in presynaptic Ca2+. J Neurosci. 2008;28(31):7765-73. Epub 2008/08/01. doi: 10.1523/JNEUROSCI.1296-08.2008

28/31/7765 [pii]. PubMed PMID: 18667608; PubMed Central PMCID: PMC2685171.
