# Supplementary material for: Chronaxie Measurements in Patterned Neuronal Cultures from Rat Hippocampus
Source: PLoS One. 2015 Jul 17;10(7):e0132577. doi: 10.1371/journal.pone.0132577 (PMC4506053; doi:10.1371/journal.pone.0132577)
Supplement: S7 Text — (DOCX) [file pone.0132577.s011.docx]

## 2D connected cultures respond isotropically

The baseline for excitation is determined on a standardly grown two dimensional culture. The axons of neurons grown in such non-patterned 2D cultures do not usually follow a particular direction. This implies that both axons and dendrites will have random orientations and that stimulation of such a culture should be directionally independent and isotropic. To verify that 2D cultures are indeed isotropic with respect to excitation by an external electric field, connected cultures (i.e. with no receptor antagonists added) were stimulated electrically. The direction of the culture with respect to the electric field was varied by rotating the electrodes manually in several different orientations, using the single-pair electrode configuration (see Fig. 1D). N=7 cultures were stimulated with a square bipolar pulse giving a field of 0.9 V/mm. Results are summarized in Fig. S3A for the cultures measured. In Fig. S3B a spherical harmonics decomposition is applied to the data (see below). Fig. S3 demonstrates that the response of 2D cultures to electric stimulation is directionally insensitive. The sphericity measure for the 2D cultures was averaged S=0.92±0.03 (n=7), which is close to a perfect circle (S=1).

## Spherical harmonics decomposition and Sphericity measure

We use the spherical harmonics defined by:


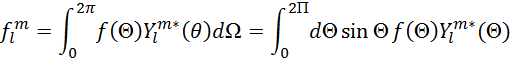
 (S1)

So that the signal can be reconstructed in the following way:


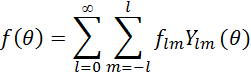
 (S2)

$Y_{lm}(\theta)$ are the Laplace spherical harmonics generally defined by:


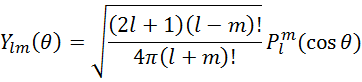
 (S3)

The $P_{l}^{m}$are Legendre polynomials.

To check that an angular distribution is isotropic and independent of angle, a “sphericity” measure was used according to (Bjorken and Brodsky 1970):

$S^{\mathrm{xy}}=\frac{\sum_{i} p_{i}^{x}p_{i}^{y}}{\sum_{i} \left| p_{i} \right|^{2}}$ (S4)

Where and ${\mathbf{p}_{\mathbf{i}}}^{\mathbf{x}}$ and ${\mathbf{p}_{\mathbf{i}}}^{\mathbf{y}}$are the x,y components. $\mathbf{S}^{\mathbf{xy}}$ is then diagonalized and its eigenvalues found. The sphericity measure is twice its smaller eigenvalue. A perfect circle would give$\mathbf{S}^{\mathbf{xy}}\mathbf{=1}$.
