# Supplementary material for: Chronaxie Measurements in Patterned Neuronal Cultures from Rat Hippocampus
Source: PLoS One. 2015 Jul 17;10(7):e0132577. doi: 10.1371/journal.pone.0132577 (PMC4506053; doi:10.1371/journal.pone.0132577)
Supplement: S8 Text — (DOCX) [file pone.0132577.s012.docx]

## Simulations - Time course of membrane charging under macro-stimulation

We began the analysis of macro-stimulation effects with finite cable in bulk solution (blue in panels A and B in S4 Fig. The observed effects can be well understood by the notion that the stimulation is physically equivalent to the simultaneous injection of equal but opposite currents at the two cable ends [[1](#_ENREF_1)]. An analytical solution for this situation is available in [[2](#_ENREF_2)] and shows that the polarization of the membrane is composed by several components with exponential time course. For cable lengths on the order of the electrotonic length constant (cable length L ≈ λ) the first component and slowest component is dominating with respect to the contributed amplitude. The time constant of this first component is

$\tau_{1}=\frac{\tau_{m}}{1+\frac{\pi^{2}\lambda^{2}}{L^{2}}}$ (SEq1).

In the example given in Fig. S4A, λ=612 µm and L=1000 µm, the effective time constant is 5.9 ms, which is far below the τ_m_=30 ms. The reason is that the current injections at the two opposing cable ends can no longer be considered independent from each other and consequently at longer times (panel A in S4 Fig, traces for 25 ms) the voltage is not decaying exponentially to zero along the cable, as it could be expected for much longer cables. Instead a near linear voltage profile develops, even for this relatively long 1mm cable.

Typical sections of the dendritic tree are even shorter. Therefore an even larger fraction of the opposing currents injected at the two cable ends cancels out directly, and the steady-state depolarization is reached much faster. In the example in panel B in S4 Fig, the effective time constant drops to 280 µs. As more of the injected current is cancelled, the depolarization of the cable’s endpoints is getting weaker (15 mV in panel B in S4 Fig vs. 60 mV in panel A in S4 Fig). An isolated soma, a structure that is essentially electrotonically compact, displayed very little depolarization in an external field (panel B in S4 Fig green), as most of the currents injected is compensated. When a soma-like structure was added to the neurite, the time course of membrane depolarization did not change much from the neurite-only case. The additional capacitance and leak attached at one end allow the current that is injected at this end to leave the cell locally. Therefore less of the current injected at the opposite, distal end is compensated for and hence the entire cable is slightly more depolarized, or, for the opposite field or direction, hyperpolarized. The effect is less pronounced the longer the cable is; the depolarization at the distal end of a very long cable (L >> λ) would not change upon addition of a soma. Already from those simple examples, it is obvious, that a combination of structures does not lead to a summation of the individual voltage profiles under the external field. Therefore, the responses of complex dendritic structures have to be studied explicitly as they cannot be derived from the simple cases studied so far. It should also be noted, that the responses of the soma and cable (panel A and B in S4 Fig), do not explain the slow and fast responses observed experimentally. The time response of the typical dendrite (200 µm) in this case would correspond to an effective time constant on the order of a few hundred microseconds and the axon response would correspond to a much larger but also slower response.

The response of a combined structure was studied next: a long cable (typical axon), a soma and a short cable (typical dendrite). The response of this system is shown in panel C in S4 Fig, it is again not the same as the linear sum of the individual elements. To aid the comparison between different charging time-courses at different locations throughout the more complex models, we renormalized the response, such that the maximum polarization has the magnitude of 1 mV. The diameter of proximal dendrites in cultured neurons does vary significantly, as is apparent from micrographs (data not shown). We therefore studied the influence of dendrite diameter on the membrane charging time course within this simple model. As the cross section of the dendrite is increased, the amplitude of the current injected at the cable ends grows proportionally and an interesting change occurs in the charging of the somatic region: the sign of the early somatic polarization is inverted. For thick dendrites, early after the stimulus onset, a hyperpolarization occurs in this region of the cell, where, based on the direction of the external field, one might have expected only depolarized potentials. This is best seen in the spatial plot for t = 0.5 ms in panel C in S4 Fig, with the time course detailed in panel D in S4 Fig, and for a longer simulation time in panel E of S4 Fig. The response at the distal end of the axon is again less affected. Another important change is the faster time response of the thicker dendrite (panel D in S4 Fig red and blue). This can be understood from Equation (SEq1) above for the dominating time constant τ_1_, because the length constant λ is proportional to the square root of the neurite diameter d.

1. Cartee LA, Plonsey R. The transient subthreshold response of spherical and cylindrical cell models to extracellular stimulation. IEEE transactions on bio-medical engineering. 1992;39(1):76-85. doi: 10.1109/10.108130.

2. Monai H, Omori T, Okada M, Inoue M, Miyakawa H, Aonishi T. An analytic solution of the cable equation predicts frequency preference of a passive shunt-end cylindrical cable in response to extracellular oscillating electric fields. Biophysical Journal. 2010;98(4):524-33. doi: 10.1016/j.bpj.2009.10.041.
