# Supplementary material for: Chronaxie Measurements in Patterned Neuronal Cultures from Rat Hippocampus
Source: PLoS One. 2015 Jul 17;10(7):e0132577. doi: 10.1371/journal.pone.0132577 (PMC4506053; doi:10.1371/journal.pone.0132577)
Supplement: S9 Text — (DOCX) [file pone.0132577.s013.docx]

## Simulations - Axial resistance and diameter determine the time response of short neurites

It is of interest to identify which parameters besides the neurite diameter exert a strong influence on the membrane charging time course. An obvious candidate would be the membrane resistance R_m_ as the membrane time constant is proportional to R_m_. However, the comparison between the results obtained with the standard parameters (blue) and a 10 times reduced membrane resistance (black) in S4 Fig shows only a marginal change in the kinetics. Although a decrease in R_m_ indeed reduces τ, another term in the denominator in Equation (SEq1) changes in parallel:

λ²= d⋅R_m_/R_i_ (SEq2)

and hence for short cables a change in R_m_ has very little influence on the kinetics of membrane charging. In contrast changing any of the other terms in Equations SEq1 and SEq2, i.e. diameter d, intracellular resistance R_i_ and cable length L, has a strong influence on the time-course (panel F in S4 Fig orange, purple, green and brown curves). This is not surprising, as reduction in L and R_i_ as well as an increase in d all reduce the electrotonic distance between the current injection points. The cancellation of the opposing currents is thus increased. Less obvious is why the amplitude of the response is largely determined by the length L of the neurite [[1](#_ENREF_1),[2](#_ENREF_2)] while changes in d and R_i_ have little influence on the amplitude. The reason is that unlike L these parameters also influence the amplitude of the injected current. Neurite diameter and length can vary greatly and are thus likely the parameters that determine the time course of membrane charging in dendrites. The key determinant for the amount of charging is of course the alignment of neurite and electric field as only the field component parallel to the neurite contributes to polarization. The components of the arbor that are oriented perpendicular to the field act as current sinks, very much as the soma attached to one end of a cable in panels A and B in S4 Fig. In contrast to the addition of the compact soma, the addition of a cable adds another, longer time constant (panel G in S4 Fig green and orange). The combination of all these factors, together with the possibly very branched and complex structure of the dendritic arbor does not allow a general statement on amplitudes and time-courses of dendritic responses. Instead, simulations with more complex models are helpful. The parameters that were identified as important determinants of the response kinetics and amplitude are rather well defined. Length and diameter can be assessed from micrographs and the axial resistance had been inferred from intracellular recordings [[3](#_ENREF_3),[4](#_ENREF_4)].

The response of the individual short cable cannot explain the time response observed for dendrites (Fig 3), which is 1.4 ms. When the time course of membrane charging is converted into a strength duration plot, the chronaxie corresponds to the stimulus duration necessary to reach 50% of the final potential. For the response of a thin 200 µm dendrite at its tip (panel B in S4 Fig) this corresponds to 350 µs. The response of single cable with a soma at its middle (dendrite-soma-dendrite, not shown) does not provide a picture very different from the results in panels B and F in S4 Fig. The symmetry of this configuration forces the potential in the middle of the structure (the soma) to be zero. Even when an axon is attached to the soma, perpendicular to the dendrite and perpendicular to the electric field, this does not noticeably change the picture, as the zero potential in the middle of the cell implies zero current flow to the axon (not shown). An asymmetric structure, on the other hand, provides a more interesting picture. The soma-dendrite combination (red in panels B and G in S4 Fig) shows that this asymmetry provides a larger effective time constant and thus a larger chronaxie as compared to the single cable. The extra leak, provided by the addition of an axon, prolongs the dendrite response adding a secondary response with a larger time constant (green). The non-stimulated axon keeps absorbing current, unbalancing the distribution of current and potentiating the voltage at the distal end of the neurite. A similar situation could be found in an axonal bend (orange in panel G in S4 Fig).

1. Cartee LA, Plonsey R (1992) The transient subthreshold response of spherical and cylindrical cell models to extracellular stimulation. IEEE transactions on bio-medical engineering 39: 76-85.

2. Rotem A, Moses E (2008) Magnetic stimulation of one-dimensional neuronal cultures. Biophysical Journal 94: 5065-5078.

3. Golding NL, Mickus TJ, Katz Y, Kath WL, Spruston N (2005) Factors mediating powerful voltage attenuation along CA1 pyramidal neuron dendrites. The Journal of Physiology 568: 69-82.

4. Schmidt-Hieber C, Jonas P, Bischofberger J (2007) Subthreshold Dendritic Signal Processing and Coincidence Detection in Dentate Gyrus Granule Cells. The Journal of Neuroscience 27: 8430-8441.
